# Effect of Indole-2-carboxylic Acid on the Self-Corrosion and Discharge Activity of Aluminum Alloy Anode in Alkaline Al–Air Battery

**DOI:** 10.3390/molecules28104193

**Published:** 2023-05-19

**Authors:** Lei Guo, Yue Huang, Alessandra Gilda Ritacca, Kai Wang, Ida Ritacco, Yan Tan, Yujie Qiang, Nabil Al-Zaqri, Wei Shi, Xingwen Zheng

**Affiliations:** 1 School of Materials and Chemical Engineering, Tongren University, Tongren 554300, China; 2 College of Materials and Metallurgy, Guizhou University, Guiyang 550025, China; yue_huang2022@163.com (Y.H.); wshi@gzu.edu.cn (W.S.); 3 Department of Life and Environmental Sciences, Polytechnic University of Marche, Via Brecce Bianche, 60131 Ancona, Ancona, Italy; a.g.ritacca@staff.univpm.it; 4 Department of Chemistry, University of Salerno, Via Giovanni Paolo II 132, 84084 Fisciano, Salerno, Italy; 5 National Center for Materials Service Safety, University of Science and Technology Beijing, Beijing 100083, China; 6 Binzhou Institute of Technology, Binzhou 256606, China; 7 Department of Chemistry, College of Science, King Saud University, P.O. Box 2455, Riyadh 11451, Saudi Arabia; nalzaqri@ksu.edu.sa; 8 Key Laboratory of Material Corrosion and Protection of Sichuan Province, Sichuan University of Science and Engineering, Zigong 643000, China

**Keywords:** Al–air battery, aluminum alloy, corrosion inhibition, indole-2-carboxylic acid, electrochemistry

## Abstract

Al–air battery has been regarded as a promising new energy source. However, the self-corrosion of aluminum anode leads to a loss of battery capacity and a decrease in battery longevity, limiting its commercial applications. Herein, indole-2-carboxylic acid (ICA) has been added to 4 M NaOH as a corrosion inhibitor. Its impact on the self-corrosion of aluminum alloy and the enhancement of the functionality of Al–air batteries at various concentrations have been investigated. X-ray photoelectron spectroscopy (XPS), attenuated total reflection Fourier transform infrared (ATR-FTIR) spectroscopy, atomic force microscopy (AFM), and scanning electron microscopy (SEM) techniques have been used to examine the compositional and morphological alterations of aluminum alloy surfaces. Electrochemical and hydrogen evolution tests showed that indole-2-carboxylic acid is an efficient corrosion inhibitor in alkaline solutions, and its impact grows with concentration. Our findings demonstrated that when the inhibitor concentration is 0.07 M, the inhibition efficiency is 54.0%, the anode utilization rises from 40.2% to 79.9%, the capacity density increases from 1197.6 to 2380.9 mAh g^−1^, and the energy density increases from 1469.9 to 2951.8 Wh kg^−1^. In addition, theoretical calculations have been performed to support the experimental results.

## 1. Introduction

Recently, metAl–air batteries have received much attention due to their exceptional performance and the escalating environmental issues caused by energy loss and environmental degradation [1,2,3]. Li-air, Mg-air, Zn-air, and Al–air batteries are the main types of aqueous metAl–air batteries powered by metals and oxygen from the air [4,5,6]. The Al–air battery stands out among these batteries due to its high theoretical energy density (8100 Wh kg^−1^), constant discharge voltage, and low cost [7,8]. The negative and positive electrodes are aluminum and airborne oxygen, respectively. Both of these substances are plentiful in the natural world and do not have any adverse effects on the ecosystem.

The electrolyte plays an important role in metAl–air batteries. Neutral or alkaline solutions are commonly utilized as electrolytes in Al–air batteries [9,10]. According to research, the aluminum anode cannot be fully utilized in neutral electrolytes as the specific energy density of the batteries would turn out constrained [11]. Aluminum anodes can be used to their fullest potential in alkaline solutions, minimizing the loss of raw resources. However, such solutions can cause aluminum anodes to self-corrode, affecting the stability of the batteries themselves and leading to a lot of hydrogen gas production [12,13]. The fundamental tenet of this parasitic reaction is outlined as follows:

(1) $$2\mathrm{Al}+6H_{2}O+2{\mathrm{OH}}^{-}\to 2\mathrm{Al}{\left(\mathrm{OH}\right)}_{4}^{-}+3H_{2}↑$$

To overcome this issue, adding a specified quantity of corrosion inhibitors to the electrolyte represents a promising solution [14,15]. It is widely accepted that the inhibitor forms a protective barrier on the surface of aluminum alloys through intermolecular bonds and/or interaction with heteroatoms and aluminum atoms, thereby reducing their self-corrosion. This will significantly impede the direct interaction of water molecules with aluminum alloys [16].

In recent years, many investigations have been carried out in recent years regarding the incorporation of corrosion inhibitors into alkaline Al–air batteries. Jiang et al. investigated the synergy between ZnO and organic acids (such as acetic acid and citric acid) to enhance the performance of alkaline Al–air batteries. They found that the battery’s voltage could reach 1.13 V in 0.3 M ZnO + 0.03 M citric acid, along with its specific capacity of 1902 mAh g^−1^ and anode utilization of 58.23% [17]. Xiang and co-workers studied the synergistic effect of 4-amino-6-hydroxy-2-mercaptopyrimidine (AHMP) and ZnO in alkaline Al–air batteries, hypothesizing a “site-directed bridging” mechanism of action. Experiments have revealed that at the optimal concentration, the mass capacity of the full battery improved from 879 to 1785 mAh g^−1^, and the anode utilization increased from 20.9% to 60% [18]. Although the addition of corrosion inhibitors to alkaline Al–air batteries has increasingly gained attention, it is extremely challenging to screen molecules that are genuinely effective for this purpose. More experiments should be conducted, notably to investigate the underlying inhibition mechanism.

**Figure 1:**
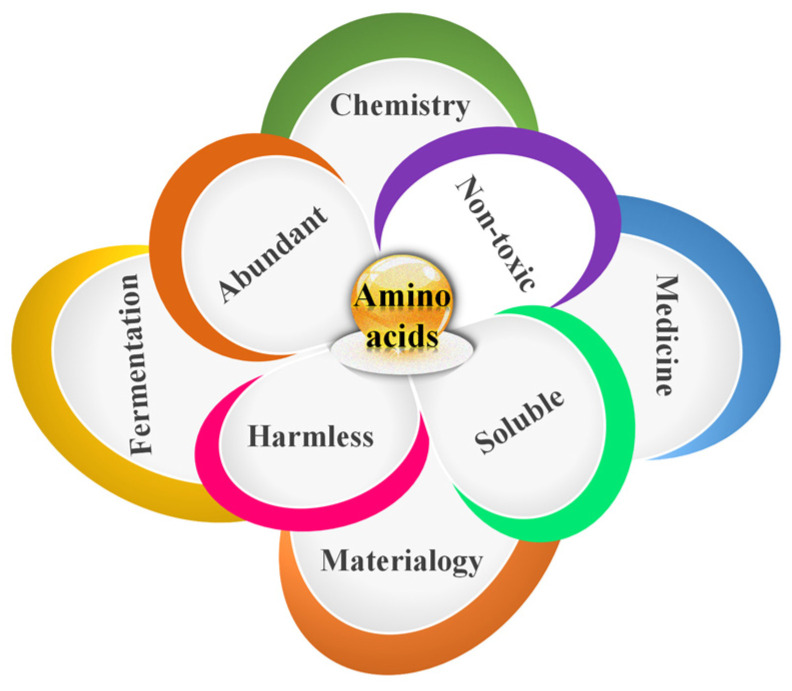
Advantages and applications of amino acids.

The present work proposes the utilization of a brand-new electrolyte additive, namely indole-2-carboxylic acid (ICA), to investigate its effects on the performance of Al–air batteries. As shown in Figure 1, as an amino acid derivative, ICA is safe, environmentally non-toxic, abundant, and inexpensive [19,20]. Due to these benefits, it has become a staple in various sectors, including the chemical, fermentation, and medical industries [21]. The distinctive −COOH functional group of ICA can efficiently bind the aluminum alloy surface, forming a barrier and preventing surface corrosion. Specifically, density functional theory (DFT) calculations were performed to disclose the anti-corrosion mechanism during the adsorption of ICA molecules on aluminum surfaces. This combined experimental and computational study offers a fresh perspective on preventing corrosion in alkaline Al–air batteries for future research.

## 2. Results and Discussion

### 2.1. OCP and PDP Analysis

**Figure 2:**
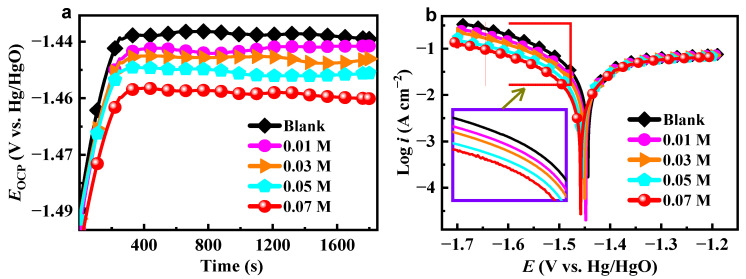
(**a**) OCP and (**b**) polarization curves of aluminum alloy in 4 M NaOH solution with and without ICA inhibitor.

The changes of OCP vs. time for aluminum alloy in 4 M NaOH solution in the absence and presence of various amounts of ICA are shown in Figure 2a. As the inhibitor concentration increases, *E*_OCP_ shifts in a negative direction, indicating that the hydrogen reduction reaction is suppressed. It suggests that ICA is primarily a cathode-type inhibitor [22]. After adding an ICA inhibitor, the PDP curves (Figure 2b) shifted to a region of decreased corrosion current density (*i*_corr_). Table 1 displays the kinetic parameters derived by extrapolating the linear segments of the anodic and cathodic branches to their point of intersection, including the corrosion potential (*E*_corr_), as well as the cathodic and anodic Tafel slopes (*β*_c_ and *β*_a_). The ICA inhibitor’s corrosion inhibition effectiveness (*η*_PDP_) was estimated as:

(2) $$\eta_{{}_{\mathrm{PDP}}}(\%)=\frac{i_{\mathrm{corr}}-i_{\mathrm{corr},\;\mathrm{inh}}}{i_{\mathrm{corr}}}\times 100$$

where *i*_corr_ and *i*_corr,inh_ represent the corrosion current densities in the absence and presence of ICA additive, respectively.

We discovered that the *i*_corr_ drops from 45.99 to 21.14 mA cm^−2^ when ICA concentration increases. When the concentration of ICA is 0.07 M, the maximum inhibitory efficiency is 54.0%. The findings indicate that a marginal modification in the *β*_c_ values substantiates the activation-controlled characteristic of hydrogen evolution. Furthermore, it can be observed that the cathode’s branching curves displayed a parallel transformation trend, suggesting that the introduction of ICA did not impact the cathodic hydrogen evolution reduction reaction mechanism.

### 2.2. EIS Measurement

**Figure 3:**
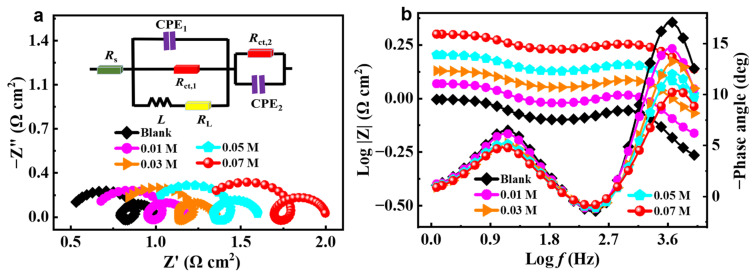
(**a**) Nyquist and (**b**) Bode curves for aluminum alloy in 4 M NaOH solution without and with different concentrations of ICA inhibitor.

Figure 3a displays Nyquist plots of the aluminum anode in 4 M NaOH without and with different concentrations of ICA inhibitor. The curves are comprised of three distinct components, namely, a low-frequency capacitance loop, a middle-frequency inductive loop, and a high-frequency capacitance loop. The capacitive arc at high frequency represents the rapid dissolution and generation between Al and Al^+^ [23]. The generation of the intermediate frequency inductive arc is attributed to the adsorption of Al(OH)_x_ on the anode surface during the process of aluminum dissolution. This process is also facilitated by the continuous adsorption of ICA molecules onto the surface of the aluminum alloy, which impedes the direct reaction between solvent molecules and the anode substrate, thereby reducing the amount of freely reactive H_2_O molecules. The low-frequency capacitive arc represents the complementary redox reaction of Al^+^→Al^3+^ [24]. An equivalent circuit (inset of Figure 3a) was used to fit the EIS data, which consists of solution resistance (*R*_s_), constant phase elements (CPE_1_, CPE_2_), inductance (*L*), the consequent inductance resistance (*R*_L_), and charge transfer resistance (*R*_ct,1_, *R*_ct,2_). The following formula is utilized to calculate the polarization resistance (*R*_p_) [25]:

(3) $$R_{p}=\frac{R_{\mathrm{ct}}{}_{,1}\times R_{L}}{R_{\mathrm{ct},1}+R_{L}}+R_{\mathrm{ct},2}$$

The impedance fitting parameters are given in Table 2. The chi-square (*χ*^2^) values were used to evaluate the accuracy of the fitted data, and the low *χ*^2^ values (approximately 10^–4^) indicate that the fitted results correlate well with the experimental data. The radius of the capacitor arc rises significantly with the addition of ICA, and the increased *R*_p_ indicates the formation of an ICA-derived barrier layer. The observed increase in the *L* value from 4.139 × 10^−5^ to 6.188 × 10^−5^ H cm^2^ indicates that, within the middle-frequency range, the inhibitor molecules exhibit a consistent tendency to displace the pre-adsorbed water molecules in the electrolytes. When ICA is added, *R*_s_ increases from 0.495 to 1.256 Ω cm^2^, which can be ascribed to the decrease in the solution conductivity. The *R*_ct,1_ and *R*_ct,2_ values increase as ICA concentration rises, whereas CPE_1_ and CPE_2_ values decrease. The phase angle constant at various ICA concentrations has been depicted on the Bode plot in Figure 3b, with two peaks indicating a dual capacitance structure during the reaction process. With increasing inhibitor concentration between 0.01 and 0.07 M, the Bode modulus increases dramatically.

### 2.3. Hydrogen Evolution Test

**Figure 4:**
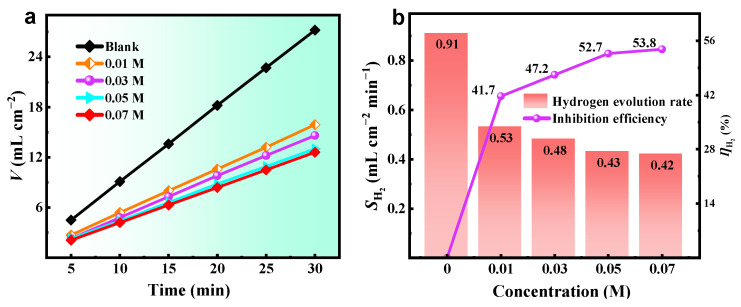
(**a**) Evolved volume of hydrogen gas when aluminum alloy soaked in blank and ICA-containing NaOH solution, and (**b**) average hydrogen evolution rate and the deduced corrosion inhibition efficiency.

The anodic self-corrosion test is conducted to directly demonstrate the anti-corrosion effect of ICA molecules on aluminum surfaces. As shown in Figure 4a, the addition of different concentrations of ICA leads to a notable reduction in the total amount of hydrogen evolution in comparison to the blank condition. The hydrogen evolution rate of aluminum decreases with the increase in the concentration of ICA, which suggests that ICA can inhibit the process of hydrogen evolution on the surface of aluminum, perhaps by squeezing the attachment sites of H_2_O molecules. The data depicted in Figure 4b indicate a decrease in *S*_H2_ from 0.91 to 0.42 mL cm^−2^ min^−1^, resulting in a *ŋ*_H2_ value of 53.8% upon the introduction of 0.07 M ICA.

### 2.4. Surface Analysis

**Figure 5:**
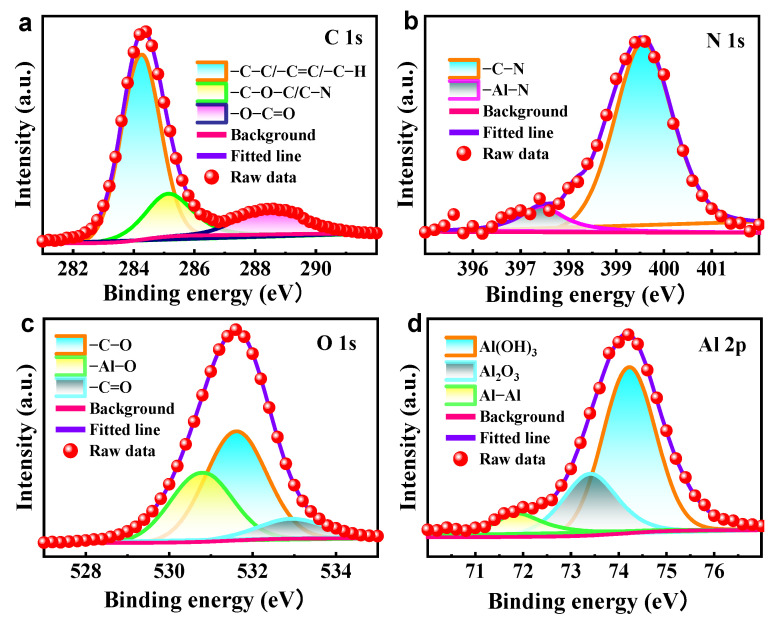
High-resolution XPS spectra of the aluminum surface after immersion in 4 M NaOH solution containing 0.07 M ICA: (**a**) C 1s, (**b**) N 1s, (**c**) O 1s, and (**d**) Al 2p.

Figure 5 displays the XPS plots of the aluminum alloy after immersion in 4 M NaOH solution with 0.07 M ICA inhibitor for 1 h. The C 1s XPS spectrum (depicted in Figure 5a) reveals three discernible peaks located at 284.2, 285.1, and 288.6 eV. These peaks have been identified as −C−C/−C=C/−C−H, −C−O−C/−C−N, and −O−C=O groups, respectively [26,27]. Two peaks with binding energies of 399.6 eV and 397.4 eV were observed in the N 1s spectrum (Figure 5b), which correspond to −C−N and Al−N, respectively [28]. The XPS pattern of O 1s is presented in Figure 5c. The observed peaks at 530.8, 531.6, and 533.0 eV are attributed to −Al−O, −C−O, and −C=O bonds, respectively. The Al 2p spectrum, as depicted in Figure 5d, exhibits distinct peaks with binding energies situated at 71.8, 73.4, and 74.2 eV. These peaks can be assigned to Al−Al, Al_2_O_3_, and Al(OH)_3_, respectively [29,30,31]. These findings indicate that the inhibitor of ICA was successfully adsorbed onto the aluminum alloy surface by covalent interactions, thereby restricting the direct interaction between the metal and the solution, leading to a reduction in corrosion.

**Figure 6:**
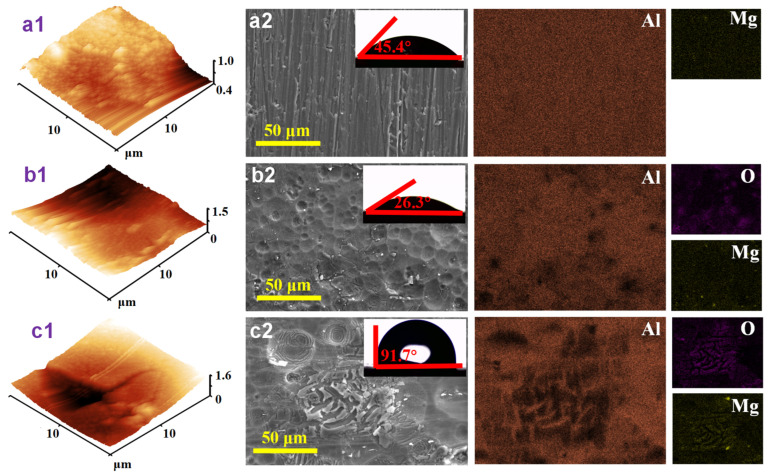
AFM, SEM, and CAM results of the aluminum alloy surface under different conditions: (**a1**,**a2**) as-polished, (**b1**,**b2**) blank, and (**c1**,**c2**) 4 M NaOH + 0.07 M ICA.

Figure 6 illustrates the surface morphology of aluminum alloy under various conditions. According to the AFM results (Figure 6a1–c1), the estimated average surface roughness values for as-polished, corroded, and protected aluminum samples are 67.1, 272.5, and 302.9 nm, respectively. After immersion in 4 M NaOH solution, the morphology of the previously smooth aluminum alloy surface is destroyed, and numerous corrosion pits appear on the surface, as shown in Figure 6a2,b2. In the inhibited condition, the ICA molecule can adhere to the metal substrate by forming a shielding film; as a consequence, the surface corrosion of the aluminum alloy is considerably reduced (Figure 6c2). In order to investigate the surface wettability of the aluminum alloy, CAM has been performed. Under blank circumstances, the contact angle on the aluminum alloy surface changes from 45.4° to 26.3°, which is attributable to the formation of a significant amount of hydrophilic corrosion products [32]. Nonetheless, the restrained approach resulted in augmented hydrophobicity of the aluminum surface due to the existence of the inhibitor film, leading to a rise in the contact angle up to 91.7°.

**Figure 7:**
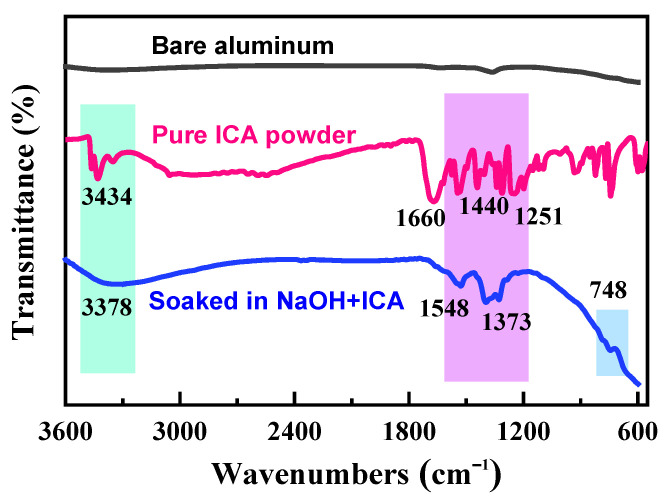
FT-IR spectra of bare aluminum, pure ICA powder, and aluminum alloy soaked in 4 M NaOH + 0.07 M ICA.

Figure 7 depicts the analysis of FT-IR spectra to reveal bonding information for various samples. Almost no peak can be seen on the surface of bare aluminum due to its non-reactive nature towards other substances. The ICA powder exhibits noteworthy peaks at 3434 and 1660 cm^−1^, which correspond to the stretching vibrations of the −NH and −COOH groups in ICA, respectively [33]. Additionally, many peaks exist within the broad spectral range of 1440–1251 cm^−1^, suggesting the skeleton vibrations of C=C and C-N, as well as the in-plane swing of the −CH group. The FT-IR spectrum of aluminum after immersion in the inhibited solution reveals the presence of COO−and C=C groups on the anode surface, and the band broadening rather than dense small peaks indicate the occurrence of typical adsorption [34]. Additionally, it should be noted that the significant peak appears at 748 cm^−1^, indicating the chemical bond between aluminum and oxygen on the anode surface [35].

### 2.5. Battery Performance

**Figure 8:**
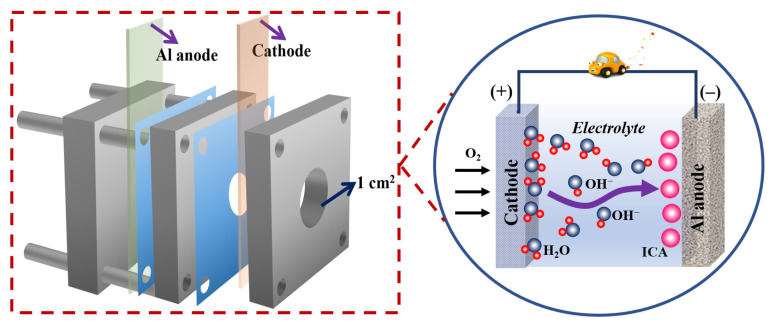
Schematic diagram of the composition and fundamentals of alkaline Al–air battery.

Figure 8 illustrates the precise structure of an Al–air full battery, which possesses an electrolyte chamber comprised of three glass plates. The self-assembled ICA film adsorbing onto the aluminum surface protects the aluminum substrate from corrosion in the alkaline medium [36]. The following are the primary processes involved in the anodic dissolution of Al–air batteries [37,38,39]:

(4) $$\mathrm{Al}+{\mathrm{OH}}^{-}\to \mathrm{Al}{\left(\mathrm{OH}\right)}_{\mathrm{ads}}+e^{-}$$

(5) $$\mathrm{Al}{\left(\mathrm{OH}\right)}_{\mathrm{ads}}+{\mathrm{OH}}^{-}\to \mathrm{Al}{\left(\mathrm{OH}\right)}_{2,\mathrm{ads}}+e^{-}$$

(6) $$\mathrm{Al}{\left(\mathrm{OH}\right)}_{2,\mathrm{ads}}+{\mathrm{OH}}^{-}\to \mathrm{Al}{\left(\mathrm{OH}\right)}_{3,\mathrm{ads}}+e^{-}\;$$

(7) $$\mathrm{Al}{\left(\mathrm{OH}\right)}_{3,\mathrm{ads}}+{\mathrm{OH}}^{-}\to \mathrm{Al}{\left(\mathrm{OH}\right)}_{4,\mathrm{ads}}^{-}$$

**Figure 9:**
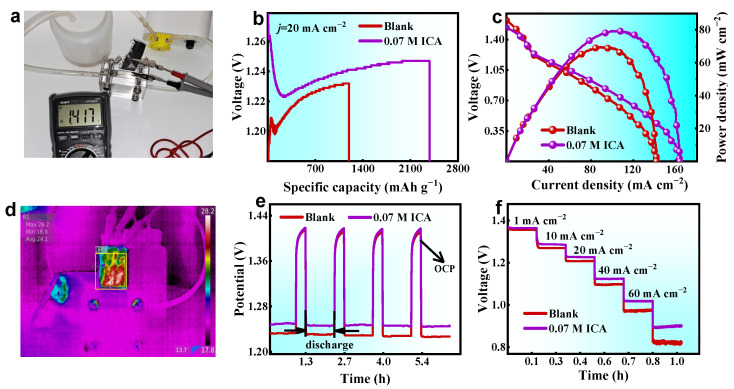
(**a**) Open-circuit voltage of the Al–air cell using ICA-based electrolyte, (**b**) specific capacity curves, (**c**) polarization curves and corresponding power density plots, (**d**) infrared thermal image, (**e**) intermittent discharge curves, and (**f**) multi-step chronopotentiometry curves.

The findings of the performance tests on the homemade Al–air battery are shown in Figure 9. The open-circuit voltage of a single cell measured with a voltmeter is 1.417 V (Figure 9a). As depicted in Figure 9b, the addition of ICA significantly increased the aluminum anode’s capacity density from 1197.6 to 2380.9 mAh g^−1^. Furthermore, the LSV analysis indicates a significant enhancement in power density subsequent to the introduction of the ICA inhibitor, as evidenced by the peak power increase from 69.22 to 78.91 mW cm^−2^ (Figure 9c). The infrared thermography results indicate that during the discharge process, the entire battery system exhibits negligible temperature fluctuations, with the highest recorded temperature being 28.2 °C (Figure 9d). To verify the durability of the electrolyte, intermittent discharge experiments simulating the practical application of the Al–air battery were conducted. As shown in Figure 9e, the voltage of the intermittent galvanostatic discharge is comparable to that of the continuous galvanostatic discharge. In addition, multi-step chronopotentiometry tests ranging from 1 to 80 mA cm^−2^ were conducted to evaluate the battery stability. Figure 9f demonstrates that the addition of ICA inhibitors leads to a notable increase in voltage at different current densities. The voltage reduction value of the Al–air battery with ICA inhibitor is significantly less than that of the blank electrolyte, indicating that ICA’s suppression of hydrogen evolution and self-corrosion contributes to the battery’s stability [40,41].

Table 3 presents the parameters acquired from the galvanostatic discharge examination. The incorporation of ICA additive results in an increase in the energy density to 2951.8 Wh kg^−1^ and a corresponding enhancement in the anode utilization up to 79.9%. Our research has ultimately demonstrated that the addition of ICA inhibitors can improve the performance of Al–air batteries.

### 2.6. Theoretical Consideration

**Figure 10:**
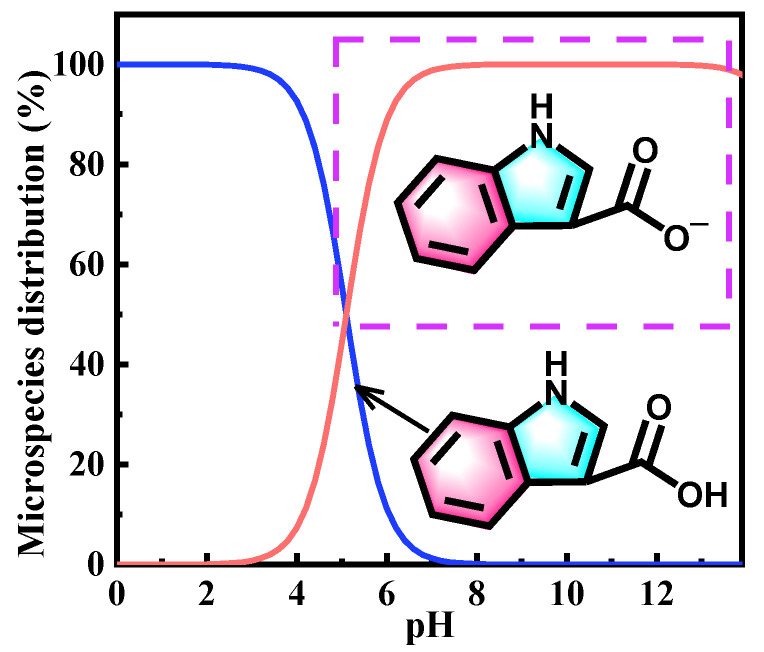
The percentage of distribution of different forms of ICA as a function of pH obtained by the Marvinsketch program.

Using the Marvinsketch program, the existing forms of ICA inhibitor at different pH levels have been analyzed, as shown in Figure 10, and it reveals that the carboxylic group has a tendency to display a deprotonation form in the extremely alkaline zone. Therefore, DFT calculations have been carried out to examine the interaction between the Al(111) surface and ICA in both its neutral and deprotonated states.

**Figure 11:**
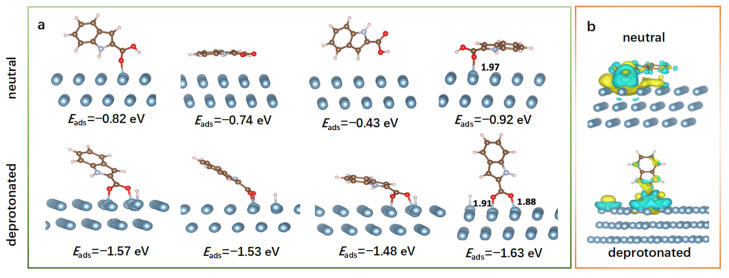
(**a**) Optimized structures of the different adsorption modes of the neutral and deprotonated ICA on Al(111) with the relative adsorption energies (*E*_ads_) reported in eV, (**b**) the charge density difference corresponding with Bader charge changes. The Al, C, O, N, and H atoms are reported in cyan, silver, red, blue, and white, respectively, and depicted in ball and stick. Bond distances are reported in angstrom (Å).

Different placements of the ICA inhibitor on the Al(111) surface have been examined, and the relative adsorption energies (*E*_ads_) for each configuration were calculated (see Figure 11a). In the most stable configuration related to the neutral form, the molecule adsorbs on Al(111) surface in a nearly parallel orientation with an *E*_ads_ of −0.92 eV, establishing π–π interactions with Al(111) through the aromatic ring of the indole group, as already observed for other metals in previous studies [42,43]. The formation of the π–π interactions results in a strong stabilization of the adsorption energy. The nature of these types of interactions is ascribed to the presence of the aromatic five-membered heterocyclic compound, such as the pyrrole portion of the indole, containing delocalized electron pairs, in which the N heteroatom has one pair of non-bonding valence shell electrons, and the benzene ring [44]. In the neutral systems, since the N atom of the pyrrole participates in aromaticity, there is no lone pair electron available for bonding interaction; therefore, together with the benzene ring, only π−π interaction modes are possible. On the contrary, O atoms have available lone pairs of electrons, and, especially, the carbonyl oxygen carries the highest negative charge. Therefore, it forms a coordination bond (1.97 Å) with the aluminum atom of the surface that facilitates the adsorption.

In the deprotonated form, the ligand interacts with the Al(111) surface in an axial orientation with an *E*_ads_ of −1.63 eV. In this case, although the π–π interactions are weak or completely lost, the adsorption energy improves compared to the neutral case due to the formation of an additional covalent bond of 1.91 Å between the deprotonated hydroxyl oxygen of the ligand and a second aluminum atom. After the interaction, the pre-existing bond between the carbonyl oxygen and the surface shortens by about 0.1 Å, becoming 1.88 Å. In the case of the deprotonated molecule, the adsorption energy (−1.63 eV) is further improved by the increase of the negative charge of the hydroxyl oxygen, which promotes the formation of two coordination bonds with aluminum atoms on the surface. The overall mechanism that describes the two adsorption phenomena can be identified as both chemisorptions since the formation of covalent bonds between the oxygens of the carbonyl group and the aluminum occurs (1.97 Å for the neutral indole and 1.91 Å and 1.88 Å for the deprotonated one), and physisorption, since electrostatic interactions between the aromatic portion of the ligand and the metal surface are established. Thus, the neutral molecule is both chemisorbed and physisorbed, whereas the deprotonated ICA is only chemisorbed through the formation of two Al-O covalent bonds [45].

The computed variation of Bader charges, calculated as the difference between the charges of the ligand atoms in free and Al(111) bound forms, indicates that during the interaction, a charge transfer occurs from the Al(111) surface to the ligand of about 0.4 and 0.5 e^−^ for neutral and deprotonated forms, respectively. During the interaction, the charge transfer concerns the carboxylic oxygens of the ligand and the aluminum atoms of the surface involved in the formation of covalent bonds. In particular, after the formation of the covalent bonds, the oxygen atoms increase their charge from 7.6 to 8.0 e^−^ both in the neutral and deprotonated form, while the aluminum atoms decrease their charge from 2.9 to 2.2 e^−^. The charge rearrangement is also confirmed by the charge density difference analysis shown in Figure 11b, where cyan and yellow colors indicate an accumulation and a depletion of charge, respectively.

## 3. Experimental

### 3.1. Materials and Reagents

The composition (in wt.%) of impurities in the selected aluminum alloy is 0.27% Fe, 0.06% Si, 2.46% Mg, 0.19% Cr, 0.10% Zn, and the remaining is Al. Prior to each experiment, the specimens’ surfaces were polished with 600, 800, 1000, and 1200 grit SiC paper, washed in ethanol, rinsed with distilled water, and dried at room temperature. The electrolyte is a 4.0 M NaOH solution with varying concentrations of ICA additive, including 0.01, 0.03, 0.05, and 0.07 M. All the used chemicals used were analytical reagents purchased from Shanghai Macklin Biochemical Co., Ltd., Shanghai, China.

### 3.2. Electrochemical Measurements

Electrochemical testing has been performed by the CHI760E electrochemical workstation using a conventional three-electrode system. Platinum sheet as the counter electrode, Hg/HgO as the reference electrode, and an aluminum alloy with a 1 cm^2^ exposed area as the working electrode. The aluminum electrode was submerged in the test solution for 30 min to generate an open circuit potential (OCP) in a steady state. Electrochemical impedance spectroscopy (EIS) measurements were performed between the frequency range of 10 kHz and 1 Hz using AC signals of 10 mV peak-to-peak amplitude at open potential (*E*_OCP_). The EIS data were fitted using ZsimpWin 3.60 software (Ametek, Inc., Berwyn, PA, USA). Potentiodynamic polarization (PDP) curves were obtained by changing the electrode potential automatically from −250 mV to + 250 mV vs. OCP at a scan rate of 1.0 mV s^−1^. In order to ensure the validity and repeatability of the electrochemical results, each experiment was conducted at least three times.

### 3.3. Hydrogen Evolution Experiment

**Figure 12:**
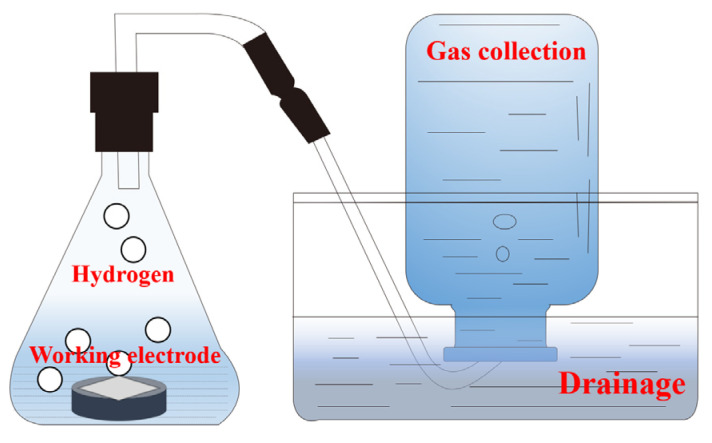
Schematic of a eudiometer used for hydrogen evolution measurement.

To evaluate the amount of hydrogen emitted from the aluminum alloy in an alkaline solution, a homemade experimental apparatus was used. As depicted in Figure 12, the apparatus consists of a conical flask, square groove, graded gas bottle, and gas-guide tube. The working electrode with a 1 cm^2^ exposed surface area was submerged in the electrolyte, and the gas produced by aluminum alloy self-corrosion was collected via a drainage technique. The data were read every five min while adding the same amount of solution to the conical flask for the experiment. Finally, the hydrogen evolution rate (*S*_H2_) and corrosion inhibition efficiency (*ŋ*_H2_) were calculated using the following equations [46,47]:

(8) $$S_{H_{2}}=\frac{V_{H_{2}}}{A\times t}$$

(9) $$\eta_{H_{2}}(\%)=\frac{S_{0}-S_{\mathrm{inh}}}{S_{0}}\times 100$$

where *A* is the exposed area of Al foil, *V*_H2_ represents the volume of collected hydrogen, and *t* indicates the soaking time. *S*_0_ and *S*_inh_ are the hydrogen evolution rates in electrolytes without and with ICA inhibitors, respectively.

### 3.4. Surface Characteristics of the Aluminum Anode

SEM (JSM-7800F) and AFM (MFP-3D-BIO) techniques have been employed to assess the surface morphology of aluminum alloy samples bathed in 4 M NaOH solution with and without a corrosion inhibitor. The elemental composition of the alloy surface that was submerged in different electrolytes was determined using energy dispersive spectroscopy (EDS). The chemical valence of the relevant element and the interaction between ICA and aluminum substrate was examined by XPS (K-Alpha) analysis. The surface bonding information for bare aluminum, ICA powder, and aluminum after immersion in the inhibited electrolyte was analyzed with the use of the ATR-FTIR spectroscopy (Nicolet iS50) method. Additionally, the contact angle measurement (CAM, JY-PHa) was implemented to quantify the surface wetting properties.

### 3.5. Battery Performance Test

The full battery performance was evaluated using a circulating pump system with a controlled flow rate of 5 mL min^−1^. The aluminum alloy was used as the battery’s negative electrode, while a commercial air electrode (acquired from Changsha Spring New Energy Technology Co., Ltd., Changsha, China) containing a MnO_2_ catalyst served as the positive electrode. The galvanostatic discharge test was conducted at a constant current density of 20 mA cm^−2^ for 1 h. The surface precipitates were then removed to determine the anode’s weight loss (Δ*m*) during discharge. The polarization curves and power-density profiles were obtained through the performance of linear sweep voltammetry (LSV) at a scan rate of 5 mV s^−1^. The intermittent discharge experiments were conducted for 20 min with an OCP, followed by 60 min with a galvanostatic discharge that cycled four times in sequence. Experiments involving multiple current steps were conducted to evaluate the stability of batteries discharged at continuously variable current densities. A thermal imaging scanner was used to detect the reaction’s temperature. The quantitative description of the battery’s performance, including specific capacity density (*Q*), anode utilization (*U*_a_), and energy density (*W*), was described through the following equations [48,49]:

(10) $$Q=\frac{It}{3600\Delta m}\;$$

(11) $$U_{a}=\frac{It}{\Delta mF/9}\times 100\%$$

(12) $$W=\frac{EIt}{3600\Delta m}\;$$

where *I* and *t* are the discharge current density and time, respectively, *F* is the Faraday constant, and *E* denotes the average discharge voltage.

### 3.6. Computational Studies

All the theoretical calculations are based on the DFT and have been performed by using the Quantum ESPRESSO computer package [50]. The exchange and correlation energy functional expressed in the Perdew–Burke–Ernzerhof (PBE) generalized gradient approximation (GGA) has been employed [51]. The spin-polarised Kohn–Sham equations have been solved in the plane-wave pseudopotential framework, with the wave function basis set and the Fourier representation of the charge density being limited by kinetic cut-offs of 50 and 500 Ry, respectively. All the atoms have been described by ultrasoft pseudopotentials [52]. In order to investigate the interaction between ICA and aluminum surface, the thermodynamically most stable Al(111) has been simulated on the basis of theoretical and experimental data [53,54]. To understand the role of the van der Waals (vdW) interactions on the ligand adsorption on the Al(111) surface, the zero damping DFT-D3 method of Grimme has been employed [55]. Al(111) surface has been modeled with periodic 6 × 5 supercell slabs consisting of four Al atomic layers separated in the direction perpendicular to the surface by 25 Å of vacuum. During the calculations, the two bottom layers of the slabs have been constrained to their equilibrium bulk-like positions, while the upper ones and the organic molecules have been fully relaxed. The Brillouin zone integration has been performed on the Γ point only. The adsorption energies (*E*_ads_) of ICA have been computed using the formula *E*_ads_(x) = *E*_surf/x_ − (*E*_surf_ + *E*_x_), where *E*_surf/x_ is the energy of the combined system (namely the surface plus the adsorbates x), *E*_surf_ is the energy of the aluminum surface, and *E*_x_ is the energy of the organic ligand in a vacuum. With this definition, a negative value of *E*_ads_ corresponds to a stable interaction between the adsorbate and the substrate. The charge analysis has been performed following Bader’s theory since the charge enclosed within the Bader volume can be considered a good approximation of the total electronic charge of an atom [56,57,58]. Charge density difference analysis has also been evaluated using the expression Δ*ρ*(r)= *ρ*_surf/x_(r) − *ρ*_surf_(r) − *ρ*_x_(r), where *ρ*_surf/x_(r), *ρ*_surf_(r), and *ρ*_x_(r) are the charge densities of the whole system, the isolated surface, and the adsorbate, respectively.

## 4. Conclusions

The outcomes demonstrate that the integration of ICA into 4 M NaOH electrolyte is a successful strategy for suppressing self-corrosion of aluminum alloy, leading to a substantial enhancement in the performance of Al–air batteries. Electrochemical experiments suggest that ICA is primarily a cathode-type inhibitor. According to surface bonding analyses and DFT simulations, the ICA molecule predominantly exists in its deprotonated form in alkaline conditions. In this case, the aluminum atom forms coordination bonds with the oxygen atoms present in the -COO^−^ group. This is crucial for the formation of the self-assembling protective film. This work paves the way for constructing durable Al–air batteries through an electrolyte regulation strategy.

## Figures and Tables

**Table 1:** Polarization parameters of aluminum alloy corrosion in the absence and presence of different concentrations of ICA in 4 M NaOH solution.

*C* (M)	*E*_corr_ (V vs. Hg/HgO)	*i*_corr_ (mA cm^−2^)	*−β*_c_ (mV dec^−1^)	*β*_a_ (mV dec^−1^)	*η*_PDP_ (%)
Blank	−1.4383	45.99	170.1	405.0	/
0.01	−1.4413	38.24	178.6	340.1	16.9
0.03	−1.4450	32.90	182.2	304.9	28.5
0.05	−1.4491	28.80	196.2	296.0	37.4
0.07	−1.4553	21.14	213.3	213.6	54.0

**Table 2:** Impedance fitting values of aluminum alloy in 4 M NaOH electrolyte without and with ICA additive.

*C* (M)	*R*_s_ (Ω cm^2^)	CPE_1_		*R*_ct,1_ (Ω cm^2^)	*L* (10^−5^ H cm^2^)	*R*_L_ (Ω cm^2^)	CPE_2_		*R*_ct,2_ (Ω cm^2^)	*R*_p_ (Ω cm^2^)	*χ*^2^ (10^−4^)
		*Y*_0_ (10^−4^ *S s^n^* cm^−2^)	*n*_1_				*Y*_0_ (*S _S_^n^* cm^−2^)	*n*_2_			
Blank	0.495	1.228	1.000	0.293	4.139	0.120	0.060	1.000	0.205	0.290	1.91
0.01	0.636	1.141	1.000	0.307	4.681	0.120	0.054	1.000	0.232	0.318	1.34
0.03	0.779	0.883	1.000	0.341	4.380	0.123	0.054	1.000	0.230	0.320	0.99
0.05	0.959	0.784	1.000	0.372	5.159	0.135	0.049	1.000	0.270	0.369	0.66
0.07	1.256	0.686	0.991	0.427	6.188	0.135	0.042	1.000	0.317	0.420	0.64

**Table 3:** Relevant parameters of Al–air battery with galvanostatic discharge (20 mA cm^−2^).

*C* (M)	Weight Loss ∆*m* (g)	Average Discharge Voltage (V)	Capacity Density (mAh g^−1^)	Energy Density (Wh kg^−1^)	*U*_a_ (%)
Blank	0.0167	1.2199	1197.6	1469.9	40.2
0.07 M ICA	0.0084	1.2398	2380.9	2951.8	79.9

## Data Availability

Data is contained within the article.
